# Association of Differential Metabolites With Small Intestinal Microflora and Maternal Outcomes in Subclinical Hypothyroidism During Pregnancy

**DOI:** 10.3389/fcimb.2021.779659

**Published:** 2022-01-07

**Authors:** Jingjing Li, Yajuan Xu, Yanjun Cai, Miao Zhang, Zongzong Sun, Yanjie Ban, Shanshan Zhai, Yingqi Hao, Qian Ouyang, Bo Wu, Mengqi Wang, Wentao Wang

**Affiliations:** Department of Obstetrics and Gynecology, The Third Affiliated Hospital of Zhengzhou University, Zhengzhou, China

**Keywords:** subclinical hypothyroidism, pregnancy, small intestinal bacteria overgrowth, metabolites, pregnancy outcomes

## Abstract

**Objective:**

To investigate the association of differential metabolites with small intestinal microflora and maternal outcomes in subclinical hypothyroidism (SCH) during pregnancy.

**Methods:**

The plasma of pregnant women in the SCH group and control group was analyzed by liquid chromatography-mass spectrometry (LC-MS), obtaining differential metabolites. Then, methane and hydrogen breath tests were performed in both groups, and basic clinical data and maternal outcome information were collected. Finally, differential metabolites were analyzed for small intestinal bacterial overgrowth (SIBO) and pregnancy outcomes using Spearman correlation analysis.

**Results:**

(1) Multivariate statistics: There were 564 different metabolites in positive ion mode and 226 different metabolites in negative ion mode. (2) The positive rate of the methane hydrogen breath test in the SCH group was higher than that in the control group (p<0.05). (3) KEGG pathway analysis revealed that differential metabolites were mainly involved in bile secretion, cholesterol metabolism, and other pathways. (4) Serum cholesterol (TC) and triglyceride (TG) levels and hypertensive disorder complicating pregnancy (HDCP) were higher in the SCH group (p<0.05), and newborn birth weight (BW) was lower than that in the control group (p<0.05). (5) SIBO was negatively correlated with glycocholic acid and BW, and positively correlated with TC. Glycocholic acid was negatively correlated with TG but positively correlated with BW. TG was positively correlated with HDCP.

**Conclusion:**

Differential metabolites in the SCH group during pregnancy were disordered with small intestinal bacteria, which may affect pregnancy outcomes, and bile acids and cholesterol may be potential biomarkers for studying their mechanism of action.

## Introduction

Subclinical hypothyroidism (SCH) in pregnancy is the most common type of hypothyroidism in pregnancy. At present, the diagnosis of hypothyroidism in pregnancy is based mainly on the examination of serum thyroid stimulating hormone (TSH) and free thyroid hormone (FT4), which lack obvious clinical symptoms (Velasco and Taylor, 2018). Although hypothyroidism during pregnancy may cause many adverse effects, such as fetal growth restriction, placental abruption, gestational hypertension, and neurodevelopmental disorders in the offspring (Fetene et al., 2017; Mahadik et al., 2020; Gietka-Czernel and Glinicki, 2021; Han et al., 2021), relevant studies on the pathogenesis of hypothyroidism and how to affect maternal and child outcomes are still scarce. Therefore, more research is needed to explore new biomarkers regarding the mechanism of action of SCH in pregnancy.

The mechanism of thyroxine metabolism is considered an important topic in the pathogenesis of SCH. Posadas-Romero et al. (2014) found that thyroid function status affects metabolism, and disturbed hepatic lipid metabolism has been shown in SCH mice (Zhang et al., 2020), but the specific mechanisms still require further investigation. Metabolomics can simultaneously respond to the changing trends of multiple metabolites in the body through systematic data screening to find biomarkers related to the disease. At present, some studies (Lauritano et al., 2007; Zhou et al., 2014; Virili and Centanni, 2015; Yao et al., 2020) show that the intestinal flora may play a role in thyroid function and that dysregulated intestinal flora can lead to hypothyroidism. Since most nutrient absorption occurs in the small intestine, it is important to understand the characteristics of the mucosa in this intestinal region (El Aidy et al., 2013). The lactulose breath test (LBT) qualitatively evaluates bacterial growth in the small intestine, with the advantages of being noninvasive, convenient, sensitive, accurate, and reproducible (Cangemi et al., 2021). Therefore, in this study, we used LC-MS to explore the changes in metabolites in the serum of pregnant women during pregnancy, and the methane hydrogen breath test evaluated the bacterial growth of the small intestine. In addition, we associated differential metabolites with SIBO-positive conditions and pregnancy outcomes and explored how SCH during pregnancy affected pregnancy outcomes.

## Subjects and Methods

### Study Subjects

Pregnant women who received regular perinatal health care in the outpatient department of the Third Affiliated Hospital of Zhengzhou University and gave birth in the hospital between July 2019 and January 2020 were randomly selected. They included 30 pregnant women with SCH (SCH group) in late pregnancy and 30 healthy pregnant women (control group) in the third trimester of pregnancy who met the inclusion criteria.

#### Inclusion Criteria

(1) The thyroid function levels of the SCH group met the diagnostic criteria in the 2017 guidelines of the American Thyroid Association for the diagnosis and management of thyroid disease during pregnancy and the postpartum (Alexander et al., 2017) and the reference-range criteria developed by the Department of Clinical Laboratory of the Third Affiliated Hospital of Zhengzhou University (11.5<FT4<22.7 pmol/L, TSH>4.0 mIU/L). The control group included pregnant women who had normal thyroid function and did not have other obstetric complications. (2) All pregnant women were in the third trimester of pregnancy.

#### Exclusion Criteria

The exclusion criteria were as follows: (1) patients aged <18 years, (2) patients who had gestational diabetes mellitus, (3) patients who tested positive for thyroid peroxidase antibody, (4) patients who had central hypothyroidism, (5) patients who were taking antithyroid drugs or thyroid hormone replacement, (6) subjects with special habits, such as taking yogurt or probiotic products every day, (7) patients with persistent poor defecation for >3 months (Jani and Marsicano, 2018), (8) patients with long-term use of antibiotics or drugs that regulate intestinal flora, (9) patients with a medical history of circulatory system, digestive system, etc., conditions, (10) patients who had been hospitalized for intestinal illness or had intestinal surgery, (11) patients whose clinical diagnosis was anxiety and depression (Slyepchenko et al., 2017), (12) patients who had used antidiarrheal drugs, probiotics, or antibiotics in the last 2 weeks, (13) subjects with abnormal stool examination results, and (14) patients who had severe liver and kidney disease.

### Specimen Collection

All pregnant women fasted for 8-12 h before blood collection. A total of 5 mL blood from the median cubital vein was collected in ethylenediaminetetraacetic acid-coated tubes. After collection, the collection tube was gently inverted four times, wrapped in aluminum foil, and temporarily stored in a 4°C refrigerator. Blood samples were centrifuged within 2 h of sample collection in a low-temperature centrifuge at 4°C and 1600×g for 10 min. Metabolite extraction was primarily performed according to previously reported methods. In short, 100 µL samples were extracted by directly adding 300 µL of precooled methanol and acetonitrile (2:1, v/v), internal standards mix 1 (IS1) and internal standards mix 2 (IS2) were added for quality control of sample preparation. After Vortex for 1 min and incubate at -20°C for 2 h, samples were centrifuged for 20 min at 4000 rpm, and the supernatant was then transferred for vacuum freeze drying. The metabolites were resuspended in 150 µL of 50% methanol and centrifuged for 30 min at 4000 rpm, and the supernatants were transferred to autosampler vials for LC-MS analysis. A quality control (QC) sample was prepared by pooling the same volume of each sample to evaluate the reproducibility of the whole LC-MS analysis.

### Data Collection

Data on age, BMI, gestational age, hypertensive disorder complicating pregnancy (HDCP), placental abruption, and serum TSH, FT4, TG, TC, LDL, and HDL levels at enrollment were collected. The neonatal Apgar scores at 1 min and 5 min, birth weight (BW), neonatal malformation, neonatal congenital methyl reduction, etc., were also collected.

### LC-MS/MS Analysis

#### Main Instruments and Reagents

The internal standard mix (IS) contains: L-Leucine-d3,L-PHENYLALANINE (13C9, 99%),L- Tryptophan-d5,Progesterone-2,3,4-13C3.

MS-grade methanol (A454-4) and acetonitrile (A996-4) were purchased from Thermo Fisher Scientific (USA). Formic acid was purchased from DIMKA (50144-50 ml, USA) and ammonium formate (17843-250G, Honeywell Fluka, USA) was obtained from Fluka. Ultrapure water was filtered through the Milli-Q system.

#### Chromatographic Conditions

The samples were analyzed on a Waters 2D UPLC (Waters, USA), coupled to a Q-Exactive mass spectrometer (Thermo Fisher Scientific, USA) with a heated electrospray ionization (HESI) source and controlled by the Xcalibur 2.3 software program (Thermo Fisher Scientific, Waltham, MA). Chromatographic separation was performed on a Waters ACQUITY UPLC BEH C18 column (1.7 μm, 2.1 mm × 100 mm, Waters, USA), and the column temperature was maintained at 45°C. The mobile phase consisted of 0.1% formic acid (A) and acetonitrile (B) in the positive mode, and in the negative mode, the mobile phase consisted of 10 mM ammonium formate (A) and acetonitrile (B). The gradient conditions were as follows: 0-1 min, 2% B; 1-9 min, 2%-98% B; 9-12 min, 98% B; 12-12.1 min, 98% B to 2% B; and 12.1-15 min, 2% B. The flow rate was 0.35 mL/min and the injection volume was 5 μL.

#### Mass Spectrometry Conditions

The mass spectrometric settings for positive/negative ionization modes were as follows: spray voltage, 3.8/-3.2 kV; sheath gas flow rate, 40 arbitrary units (arb); aux gas flow rate, 10 arb; aux gas heater temperature, 350°C; capillary temperature, 320°C. The full scan range was 70 - 1050 m/z with a resolution of 70,000, and the automatic gain control (AGC) target for MS acquisitions was set to 3e6 with a maximum ion injection time of 100 ms. The top 3 precursors were selected for subsequent MSMS fragmentation with a maximum ion injection time of 50 ms and resolution of 30,000, the AGC was 1e5. The stepped normalized collision energy was set to 20, 40, and 60 eV.

In order to provide more reliable experimental results during instrument testing, the samples are randomly ordered to reduce system errors. A QC sample is interspersed for every 10 samples.

### Methane Hydrogen Breath Test

The intake of foods that are prone to gas production, and rich in cellulose and carbohydrates, such as dairy products, soy products, artificially sweetened foods and beverages, alcohol-containing preparations, wheat noodles, high cellulose vegetables, etc., were prohibited within the 24 h before the test, while cooked rice, beef, baked skinless chicken, eggs, and sugar-free water were allowed. Within the 12 h before the test, subjects were only allowed to drink a small amount of boiled water. On the test day, smoking (including second-hand smoke) was prohibited, subjects should brush their teeth, stay awake and quiet, and avoid intense activities (Rezaie et al., 2017). The level of hydrogen and methane in the breath samples was measured using the BreathTracker SC (QuinTron, USA). The baseline level of hydrogen and methane in the fasting state was measured after calibration with standard gas. A total of 10 g of lactulose oral solution (Beijing Hanmi Pharmaceutical Co., Ltd.) was dissolved in about 239 ml of warm water, which was quickly consumed by the subject, and the breath of the subject was tested every 20 min for 120 min (Cohen-Mekelburg et al., 2018). The level of hydrogen and methane at each time point was measured and recorded, based on which the Time-Abundance curves for hydrogen and methane were generated.

#### Diagnostic Method for SIBO

The breath test results were analyzed as follows (Lee et al., 2013; 2014; Kasir et al., 2016): (1) an increase of ≥ 20 ppm from baseline in hydrogen by 90 min was considered a positive test to suggest the presence of SIBO; (2) if the methane concentration was higher than the fasting baseline value by 10 ppm within 90 min of the breath test, SIBO was considered positive; and (3) if the hydrogen and methane concentration did not reach the above values, the sum of the two was higher than the sum of the fasting baseline values of hydrogen and the methane concentration was more than 15 ppm within 90 min of the breath test, SIBO was considered positive.

### Statistical Analysis

LC-MS/MS raw data (raw file) were imported into Compound Discoverer 3.1 (Thermo Fisher Scientific, USA) for data processing for peak extraction, retention time correction within and between groups, and metabolite identification, and the compound molecular weight, retention time, peak area, and identification results were exported. Later, metaX was imported for data preprocessing, and the data were corrected by the QC-RLSC method. Differential metabolites between groups were screened using a combination of multiple statistical analyses (PCA and PLS-DA) and univariate analysis [multiple of difference change (fold change, FC) and T test (Student’s t test)]. For the metabolic pathway enrichment analysis of differential metabolites based on the KEGG database, the metabolic pathway of p value <0.05 was significantly enriched for differential metabolites. This study used SPSS software version 25.0 to process the basic information of pregnant women. The normal distribution is described by the mean ± standard deviation (χ ± s), and an independent sample t test was used to compare differences between groups. Categorical variables are described as frequency, and the differences between groups were determined by the chi-squared test. Correlation analysis was performed using the Spearman analysis method.

## Results

### General Clinical Data and Pregnancy Outcomes

This study enrolled 30 patients in the SCH group and 30 patients at the same stage of pregnancy in the control group. **Table 1** provides the general clinical data and pregnancy outcomes of the subjects in the two groups. There was no significant difference between the two pregnancy groups in age, prepregnancy BMI, week gestation, FT4, high-density lipoprotein (HDL), Apgar scores at 1 and 5 min, placental abruption, neonatal malformations, or neonatal congenital hypothyroidism. However, the serum TSH, TC, TG, and LDL levels, newborn BW levels, and occurrence of HDCP were significantly different between the two groups (p<0.05).

**Table 1:** Comparison of general clinical data between the SCH group and the control group.

	SCH group	Control group	P value
Maternal age, year	30.40 ± 4.01	29.67 ± 4.06	0.485
BMI, Kg/m^2^	21.72 ± 2.75	21.79 ± 2.71	0.917
Week of pregnancy, week	37.7 ± 1.86	37.53 ± 1.55	0.707
TSH, mIU/L	4.84 ± 0.86	1.86 ± 0.82	0.000*
FT4, pmol/L	12.51 ± 1.10	12.26 ± 0.81	0.319
TG, mmol/L	2.54 ± 0.37	1.83 ± 0.53	0.000*
TC, mmol/L	6.56 ± 0.89	4.89 ± 0.90	0.000*
LDL, mmol/L	4.35 ± 0.76	2.95 ± 0.75	0.000*
HDL, mmol/L	2.06 ± 0.31	1.94 ± 0.34	0.152
Placental abruption, n	1	0	0.317
HDCP, n	6	1	0.046*
Apgar scores			
1 min	9.73 ± 0.58	9.83 ± 0.38	0.434
5 min	9.87 ± 0.35	9.90 ± 0.31	0.694
BW, g	3176.54 ± 298.11	3425.81 ± 294.31	0.002*
Neonatal malformations, n	0	0	1.000
Neonatal congenital hypothyroidism, n	0	0	1.000

*P < 0.05 was considered statistically significant.

### Multivariate Statistical Analysis of the Metabolomics Data

**Figure 1:**
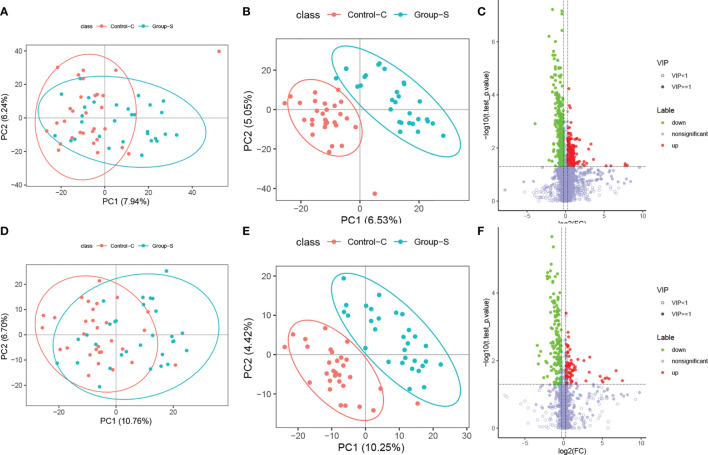
Differential metabolites analysis for the SCH group and the control group. **(A)** PCA plot in positive ionic mode; **(B)** PLD-DA plot in positive ionic mode; **(C)** Volcano plot in positive ionic mode; **(D)** PCA plot in negative ionic mode; **(E)** PLD-DA plot in negative ionic mode; **(F)** Volcano plot in negative ionic mode.

Unsupervised PCA was performed on the experimental and control samples, with the first two principal components PC1 and PC2 being PC1 (7.94%) and PC2 (6.24%) in positive ion mode (**Figure 1A**) and PC1 (10.76%) and PC2 (6.70%) in negative ion mode (**Figure 1D**). In the positive-negative ion mode, most of the sample points of the two groups overlapped in the two-dimensional PCA point mode, but there were still differences in the first principal component and some anomalous points, indicating differences in experimental and control metabolism.

To further validate the significant differences between the experimental and control groups, the two data groups were remodeled and analyzed using a supervised PLS-DA multivariate approach. The scores of the first two major components, PC1-PC2, obtained in positive and negative ion mode were PC1 (6.53%), PC2 (5.05%), PC1 (10.25%), and PC2 (4.42%), as shown in **Figures 1B, E**, respectively. In positive-negative ion mode, the sample points in the two groups were completely separated in two-dimensional PCA point mode, indicating that the metabolism between the experimental group was different from that of the control group.

By calculating the strength of the importance of variable projection significance (variable important for the projection, VIP), a VIP greater than 1 indicates that the variable has a significant effect on the discrimination of the sample categories. Conditions for differential metabolite screening:

1) The VIP of the first two principal components in the PLS-DA model ≥1, 2) FC≥1.2 or ≤0.83, and 3) p value <0.05. The volcano plots take log2 (FC) as the abscissa and log10 (P values) as the ordinate, and the final positive and negative ion patterns are shown in **Figures 1C, F**. Multivariate statistical analysis found a total of 564 differential metabolites in positive ionic mode, with 247 upregulated and 317 downregulated, and 226 differential metabolites in negative ionic mode, with 83 upregulated and 143 downregulated. Each dot in the figure represents one metabolite, red dots represent upregulated differential metabolites, green dots represent downregulated metabolites, and gray dots represent nondifferent metabolites.

### Methane Hydrogen Breath Test Results

**Figure 2:**
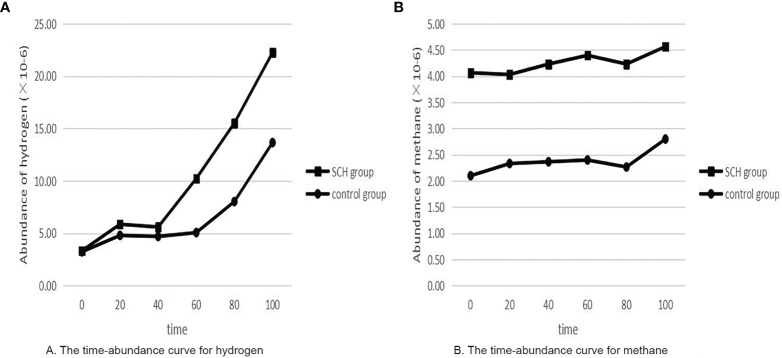
The time-abundance curve for hydrogen and methane. **(A)** The time-abundance curve for hydrogen; **(B)** The time-abundance curve for methane.

**Table 2** shows that the SCH group had significantly higher SIBO-positive rates than the control pregnant women (p<0.05). There was no significant difference in the pure hydrogen positive rate (p> 0.05). Pure methane positivity and hydroxide positivity were statistically significant (p<0.05). The mean expiratory hydrogen abundance (**Figure 2A**) and mean expiratory methane abundance (**Figure 2B**) were significantly higher in the experimental group than in the control group.

**Table 2:** The rate of SIBO positive, pure hydrogen-positive, pure methane-positive, and hydrogen-methane positive between SCH group and the control group.

	SIBO+	Hydrogen+	Methane+	Hydrogen-methane+
SCH group	22 (73.3%)	8 (26.7%)	4 (13.3%)	10 (33.3%)
Control group	11 (36.7%)	8 (26.7%)	0 (0.0%)	3 (10.0%)
X^2^	8.148	0.000	4.286	4.812
p	**0.004**	1.000	**0.038**	**0.028**

**P < 0.05** was considered statistically significant.

### Pathways Analyses

**Figure 3:**
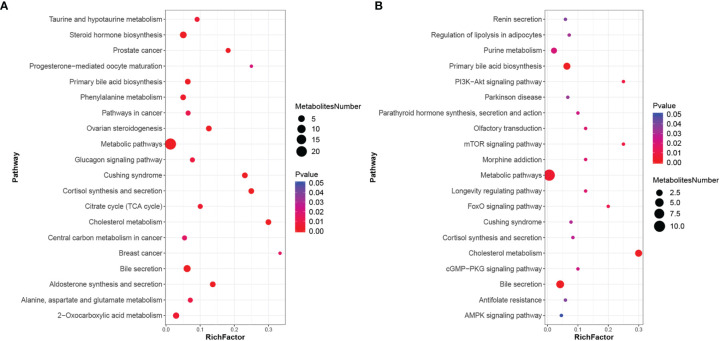
KEGG enrichment scatter plot. **(A)** KEGG enrichment scatter plot in positive ionic mode; **(B)** KEGG enrichment scatter plot in negative ionic mode.

Using metabolic pathway enrichment analysis of differential metabolites based on the KEGG database, the metabolic pathway with a p value <0.05 was significantly enriched for differential metabolites, and bubble plots of pathways with significantly enriched differential metabolites are shown in **Figure 3**. Differential metabolites involve multiple pathways, mainly bile secretion, cholesterol metabolism, primary bile acid synthesis, etc.

### Correlation Analysis of SIBO With Metabolites and Clinical Data

The differential metabolite glycocholic acid, the most divergent pathway in the KEGG metabolic pathway, was selected for correlation analysis with SIBO and clinical data, as shown in **Table 3**. SIBO was negatively correlated with glycocholic acid levels, BMI, and BW and positively associated with TSH and TC levels. Glycocholic acid was negatively correlated with TSH and TG levels but positively correlated with BW. TG was positively correlated with HDCP.

**Table 3:** Correlation analysis of SIBO with metabolites and clinical data.

Factor 1	Factor 2	r	p
SIBO	Glycocholicacid	-0.370	**0.004**
SIBO	BMI	-0.399	**0.002**
SIBO	TSH	0.377	**0.003**
SIBO	TC	0.352	**0.006**
SIBO	BW	-0.462	**0.000**
Glycocholicacid	BW	0.354	**0.006**
Glycocholicacid	TSH	-0.297	**0.021**
Glycocholicacid	TG	-0.384	**0.002**
TG	HDCP	0.514	**0.000**

**P < 0.05** was considered statistically significant.

## Discussion

Thyroid disease is the second largest endocrine disease after diabetes in pregnant women, and subclinical hypothyroidism during pregnancy has the highest incidence of thyroid disease. The intestinal flora is considered to be the largest endocrine organ in the human body, directly participating in a variety of metabolic processes of the body and affecting the function of the thyroid and other organs (Virili and Centanni, 2017; Li et al., 2021). Most current studies use 16S rRNA technology to detect the intestinal microbiota characteristics of diseases, and research on the correlation between them is scarce. In this study, metabolomics combined with the methane hydrogen breath test was used to evaluate the metabolism of hypothyroidism during pregnancy and the occurrence of SIBO.

Metabolic multiple statistical analysis found 564 differential metabolites in the experimental and control pregnant women in the positive ion mode and 226 in the negative ion mode. In both positive and negative ion patterns, we found decreased bile acid levels in the experimental group compared with control pregnant women, and a negative association with TSH by glycocholic acid. Wu et al. (2005) and Song et al. (2016) have similarly found TSH to be an important regulator of BA homeostasis, with decreased bile acid levels in hypothyroidism patients. Therefore, we consider its possible mechanism as follows: (1) Alternative pathways such as sulfidation and glucuronidation in the liver may function in thyroid hormone metabolism in peripheral tissues, and these combinations can ester phenolic hydroxyl groups with sulfate or glucuronate, which increases the water solubility of iodothyronine, resulting in elevated bile and kidney clearance and reduced intestinal absorption. (2) TSH can inhibit the activity of cholesterol 7 α-hydroxylase (CYP7A1) through the TSH receptor (TSHR) in the liver, leading to reduced bile acid (BA) levels.

In this study, the SIBO-positive rate was higher in the experimental group than in the control pregnant women. When SIBO occurs, abnormal bacteria in the small intestine metabolize carbohydrates that produce hydrogen, methane, and carbon dioxide, transported by passive diffusion to lung capillaries and discharged from the body, whose contents can be detected by chromatography (Dabritz et al., 2014). However, our study found no significant difference in the incidence of SIBO by hydrogen abundance alone. Therefore, the addition of methane and methane with hydrogen is more accurate for assessing the incidence of SIBO in pregnant women. Of course, the reason why there is no obvious difference between hydrogen may be that hydrogen in the gut is consumed to produce methane and H_2_S gases. However, the line chart of this experiment still shows the abundance of hydrogen and methane in the exhaled gas in the experimental group compared with the control group, and TSH was positively associated with SIBO. This shows that small intestinal bacteria are disordered in pregnant women during pregnancy and that the abnormal growth of bacteria produces hydrogen and methane in the small intestine.

Using KEGG metabolic pathway analysis of differential metabolites, we found that the two pathways of bile acid secretion and cholesterol metabolism were most significantly different in both positive and negative ion modes, and both pathways contained glycocholic acid, taurochenodeoxycholic acid, and glycochenodeoxycholate. We found that SIBO was negatively correlated with glycocholic acid. Intestinal bacteria are considered the largest endocrine organ in the body. Animal experiments (Swann et al., 2011) indicate significant differences in the composition of bile acids and the gene expression profiles involved in bile acid synthesis, binding, and reabsorption in sterile mice and mice with normal flora. Disruption of the gut flora reduces the number of bacteria producing secondary bile acids and reduces the levels of secondary bile acids (cholic acid and deoxycholic acid) (Ge et al., 2018). In addition, bile acids also have certain bacterial toxicity. In addition to inhibiting methanogens, they can inhibit other hydrogen-consuming bacteria, which can affect the growth rate of bacteria and change the levels of genes related to intestinal bacteria involved in lipid and amino acid metabolism. Thus, the interaction of bile acids with the gut flora may contribute to the metabolic disorder of SCH in pregnancy.

Regarding pregnancy outcome, our study found the following: (1) The incidence of gestational hypertension was higher in the experimental group and had a positive association with TG levels. In the clinical data analysis, we found that cholesterol and triglyceride levels in the women in the experimental group were significantly higher than those in the control pregnant women. Delitala et al. (2020) and Zhou et al. (2020) found the same results. The decrease in bile acid levels in pregnant women in the SCH group during pregnancy may increase triglyceride levels by raising the expression of sterol response element binding protein 1c in the liver and lipid accumulation in blood vessels, leading to atherosclerosis and inducing hypertension. (2) Neonatal birth weight can be affected by a variety of factors. The BW in the SCH group was significantly lower than that in the control group and was negatively associated with SIBO. When SIBO occurs, the gut flora is disordered, and small intestinal epithelial cells (IECs) are disrupted, thereby affecting individual absorption of carbohydrates, proteins, and lipids, and increasing nutrient competition by the presence of bacteria (Quigley and Quera, 2006). Fetuses acquire nutrients from the maternal blood so that microbial-induced bacterial components or metabolites can be effectively transferred to the fetus within the mother. (3) BW was positively correlated with bile acid. (Song et al., 2021) also found the same results. Bile acids, an essential metabolic molecule for dietary lipids and the absorption and metabolism of lipid-soluble vitamins, can bind to receptors (FXR and TGR5) to stimulate GLP-1 and PYY secretion (Sonne et al., 2014; Xie et al., 2021), affecting energy intake and absorption. Therefore, bile acids may affect gastrin secretion through FXR and TGR5 receptors, affecting energy absorption in pregnant women and in turn affecting neonatal birth weight.

Due to the difficulty of obtaining samples, previous studies usually conducted 16S rRNA sequencing to assess intestinal microflora characteristics without exploring pure small intestinal bacterial characteristics. In this experiment, the methane hydrogen breath test was used to evaluate the small intestinal flora of pregnant women with hypothyroidism, and the correlation between the occurrence of SIBO in the methane hydrogen breath test of pregnant women with subclinical hypothyroidism and the differential metabolites obtained by metabolomics was analyzed. The relationship between SCH during pregnancy and small intestinal bacteria and metabolism was preliminarily discussed to provide ideas for the study of the specific mechanism between SCH during pregnancy and intestinal bacteria and metabolism. However, this study also has limitations, namely, a small sample size, the enrolled pregnant women have certain regional limitations, and their different diets may have some impact on the experiment.

In conclusion, this study explored the correlation between the metabolism of SCH during pregnancy and intestinal flora through discussing the differences in metabolite levels and SIBO incidence between SCH and normal pregnant women and revealed that TSH may reduce bile acid levels through alternative ways or by binding with TSHR in the liver. Bile acids can affect the body’s lipid metabolism by increasing the expression of sterol response element binding protein 1c in the liver, increasing the triglyceride level, reducing the conversion of cholesterol to bile acids and increasing the cholesterol level, which may increase the risk of pregnant women suffering from hypertensive disorder complicating pregnancy. Due to the bacterial toxicity of bile acid, inhibiting methanogens, leading to the occurrence of SIBO, affects the absorption of nutrients and may then affect the newborn birth weight. Therefore, bile acids and cholesterol may be new biomarkers for studying the mechanisms of subclinical hypothyroidism during pregnancy, which in turn can improve pregnancy outcome.

## Data Availability Statement

The raw data supporting the conclusions of this article will be made available by the authors, without undue reservation.

## Ethics Statement

The studies involving human participants were reviewed and approved by the Ethics Committee of the Third Affiliated Hospital of Zhengzhou University. The patients/participants provided their written informed consent to participate in this study. Written informed consent was obtained from the individual(s) for the publication of any potentially identifiable images or data included in this article.

## Author Contributions

YX conceived and designed research. YX, YC, MZ, ZS, YB, SZ, YH, QO, BW, MW, and WW performed experiments. JL, YC, and MZ analyzed data. JL, YX, YC, MZ, ZS, and YB, interpreted results of experiments. JL prepared figures. JL drafted the manuscript. JL, YX, and MZ edited and revised the manuscript. All authors contributed to the article and approved the submitted version.

## Funding

This work was supported by Henan Science and Technology Department (Item No. SBGJ202002090).

## Conflict of Interest

The authors declare that the research was conducted in the absence of any commercial or financial relationships that could be construed as a potential conflict of interest.

## Publisher’s Note

All claims expressed in this article are solely those of the authors and do not necessarily represent those of their affiliated organizations, or those of the publisher, the editors and the reviewers. Any product that may be evaluated in this article, or claim that may be made by its manufacturer, is not guaranteed or endorsed by the publisher.

## References

[B1] Alexander E. K. Pearce E. N. Brent G. A. Brown R. S. Chen H. Dosiou C.. (2017). 2017 Guidelines of the American Thyroid Association for the Diagnosis and Management of Thyroid Disease During Pregnancy and the Postpartum. Thyroid 27, 315–389. doi: 10.1089/thy.2016.0457 28056690

[B2] Cangemi D. J. Lacy B. E. Wise J. (2021). Diagnosing Small Intestinal Bacterial Overgrowth: A Comparison of Lactulose Breath Tests to Small Bowel Aspirates. Dig Dis. Sci. 66, 2042–2050. doi: 10.1007/s10620-020-06484-z 32681227

[B3] Cohen-Mekelburg S. Tafesh Z. Coburn E. Weg R. Malik N. Webb C.. (2018). Testing and Treating Small Intestinal Bacterial Overgrowth Reduces Symptoms in Patients With Inflammatory Bowel Disease. Dig Dis. Sci. 63, 2439–2444. doi: 10.1007/s10620-018-5109-1 29761252

[B4] Dabritz J. Muhlbauer M. Domagk D. Voos N. Hennebohl G. Siemer M. L.. (2014). Significance of Hydrogen Breath Tests in Children With Suspected Carbohydrate Malabsorption. BMC Pediatr. 14, 59. doi: 10.1186/1471-2431-14-59 24575947 PMC3975941

[B5] Delitala A. P. Scuteri A. Maioli M. Casu G. Merella P. Fanciulli G. (2020). Effect of rhTSH on Lipids. J. Clin. Med. 9, 515–561. doi: 10.3390/jcm9020515 PMC7073530 32074945

[B6] El Aidy S. Merrifield C. A. Derrien M. Van Baarlen P. Hooiveld G. Levenez F.. (2013). The Gut Microbiota Elicits a Profound Metabolic Reorientation in the Mouse Jejunal Mucosa During Conventionalisation. Gut 62, 1306–1314. doi: 10.1136/gutjnl-2011-301955 22722618

[B7] Fetene D. M. Betts K. S. Alati R. (2017). Mechanisms in Endocrinology: Maternal Thyroid Dysfunction During Pregnancy and Behavioural and Psychiatric Disorders of Children: A Systematic Review. Eur. J. Endocrinol. 177, R261–R273. doi: 10.1530/EJE-16-0860 28982961

[B8] Ge X. Pan J. Liu Y. Wang H. Zhou W. Wang X. (2018). Intestinal Crosstalk Between Microbiota and Serotonin and its Impact on Gut Motility. Curr. Pharm. Biotechnol. 19, 190–195. doi: 10.2174/1389201019666180528094202 29804531

[B9] Gietka-Czernel M. Glinicki P. (2021). Subclinical Hypothyroidism in Pregnancy: Controversies on Diagnosis and Treatment. Pol. Arch. Intern. Med. 131, 266–275. doi: 10.20452/pamw.15626. 32975922

[B10] Han L. Ma Y. Liang Z. Chen D. (2021). Laboratory Characteristics Analysis of the Efficacy of Levothyroxine on Subclinical Hypothyroidism During Pregnancy: A Single-Center Retrospective Study. Bioengineered 12, 4183–4190. doi: 10.1080/21655979.2021.1955589 34288808 PMC8806776

[B11] Jani B. Marsicano E. (2018). Constipation: Evaluation and Management. Mo Med. 115, 236–240. 30228729 PMC6140151

[B12] Kasir R. Zakko S. Zakko P. Adler M. Lee A. Dhingra S.. (2016). Predicting a Response to Antibiotics in Patients With the Irritable Bowel Syndrome. Dig Dis. Sci. 61, 846–851. doi: 10.1007/s10620-015-3872-9 26362282

[B13] Lauritano E. C. Bilotta A. L. Gabrielli M. Scarpellini E. Lupascu A. Laginestra A.. (2007). Association Between Hypothyroidism and Small Intestinal Bacterial Overgrowth. J. Clin. Endocrinol. Metab. 92, 4180–4184. doi: 10.1210/jc.2007-0606 17698907

[B14] Lee K. N. Lee O. Y. Koh D. H. Sohn W. Lee S. P. Jun D. W.. (2013). Association Between Symptoms of Irritable Bowel Syndrome and Methane and Hydrogen on Lactulose Breath Test. J. Korean Med. Sci. 28, 901–907. doi: 10.3346/jkms.2013.28.6.901 23772156 PMC3678008

[B15] Li A. Li T. Gao X. Yan H. Chen J. Huang M.. (2021). Gut Microbiome Alterations in Patients With Thyroid Nodules. Front. Cell Infect. Microbiol. 11, 643968. doi: 10.3389/fcimb.2021.643968 33791245 PMC8005713

[B16] Mahadik K. Choudhary P. Roy P. K. (2020). Study of Thyroid Function in Pregnancy, its Feto-Maternal Outcome; a Prospective Observational Study. BMC Pregnancy Childbirth 20, 769. doi: 10.1186/s12884-020-03448-z 33302910 PMC7726876

[B17] Posadas-Romero C. Jorge-Galarza E. Posadas-Sanchez R. Acuna-Valerio J. Juarez-Rojas J. G. Kimura-Hayama E.. (2014). Fatty Liver Largely Explains Associations of Subclinical Hypothyroidism With Insulin Resistance, Metabolic Syndrome, and Subclinical Coronary Atherosclerosis. Eur. J. Endocrinol. 171, 319–325. doi: 10.1530/EJE-14-0150 25053728

[B18] Quigley E. M. Quera R. (2006). Small Intestinal Bacterial Overgrowth: Roles of Antibiotics, Prebiotics, and Probiotics. Gastroenterology 130, S78–S90. doi: 10.1053/j.gastro.2005.11.046 16473077

[B19] Rezaie A. Buresi M. Lembo A. Lin H. McCallum R. Rao S.. (2017). Hydrogen and Methane-Based Breath Testing in Gastrointestinal Disorders: The North American Consensus. Am. J. Gastroenterol. 112 (5), 775–784. doi: 10.1038/ajg.2017.46 28323273 PMC5418558

[B20] Slyepchenko A. Maes M. Jacka F. N. Kohler C. A. Barichello T. Mcintyre R. S.. (2017). Gut Microbiota, Bacterial Translocation, and Interactions With Diet: Pathophysiological Links Between Major Depressive Disorder and Non-Communicable Medical Comorbidities. Psychother. Psychosom 86, 31–46. doi: 10.1159/000448957 27884012

[B21] Song F. Chen Y. Chen L. Li H. Cheng X. Wu W. (2021). Association of Elevated Maternal Serum Total Bile Acids With Low Birth Weight and Intrauterine Fetal Growth Restriction. JAMA Netw. Open 4, e2117409. doi: 10.1001/jamanetworkopen.2021.17409 34279647 PMC8290304

[B22] Song Y. Zhao M. Zhang H. Zhang X. Zhao J. Xu J.. (2016). Thyroid-Stimulating Hormone Levels Are Inversely Associated With Serum Total Bile Acid Levels: A Cross-Sectional Study. Endocr. Pract. 22, 420–426. doi: 10.4158/EP15844.OR 26606535

[B23] Sonne D. P. Rehfeld J. F. Holst J. J. Vilsboll T. Knop F. K. (2014). Postprandial Gallbladder Emptying in Patients With Type 2 Diabetes: Potential Implications for Bile-Induced Secretion of Glucagon-Like Peptide 1. Eur. J. Endocrinol. 171, 407–419. doi: 10.1530/EJE-14-0309 24986531

[B24] Swann J. R. Want E. J. Geier F. M. Spagou K. Wilson I. D. Sidaway J. E.. (2011). Systemic Gut Microbial Modulation of Bile Acid Metabolism in Host Tissue Compartments. Proc. Natl. Acad. Sci. U. S. A. 108 (Suppl 1), 4523–4530. doi: 10.1073/pnas.1006734107 20837534 PMC3063584

[B25] Velasco I. Taylor P. (2018). Identifying and Treating Subclinical Thyroid Dysfunction in Pregnancy: Emerging Controversies. Eur. J. Endocrinol. 178, D1–D12. doi: 10.1530/EJE-17-0598 29070512

[B26] Virili C. Centanni M. (2015). Does Microbiota Composition Affect Thyroid Homeostasis? Endocrine 49, 583–587. doi: 10.1007/s12020-014-0509-2 25516464

[B27] Virili C. Centanni M. (2017). "With a Little Help From My Friends" - The Role of Microbiota in Thyroid Hormone Metabolism and Enterohepatic Recycling. Mol. Cell Endocrinol. 458, 39–43. doi: 10.1016/j.mce.2017.01.053 28167127

[B28] World Health Organization. (2013). Diabetes. Available at: http://who.int/diabetes/en [Accessed January 1 ,2013].

[B29] Wu S. Y. Green W. L. Huang W. S. Hays M. T. Chopra I. J. (2005). Alternate Pathways of Thyroid Hormone Metabolism. Thyroid 15, 943–958. doi: 10.1089/thy.2005.15.943 16131336

[B30] Xie C. Huang W. Young R. L. Jones K. L. Horowitz M. Rayner C. K.. (2021). Role of Bile Acids in the Regulation of Food Intake, and Their Dysregulation in Metabolic Disease. Nutrients 13, 1104–1119. doi: 10.3390/nu13041104 33800566 PMC8066182

[B31] Yao Z. Zhao M. Gong Y. Chen W. Wang Q. Fu Y.. (2020). Relation of Gut Microbes and L-Thyroxine Through Altered Thyroxine Metabolism in Subclinical Hypothyroidism Subjects. Front. Cell Infect. Microbiol. 10, 495. doi: 10.3389/fcimb.2020.00495 33072620 PMC7531258

[B32] Zhang L. Wu K. Bo T. Zhou L. Gao L. Zhou X.. (2020). Integrated microRNA and Proteome Analysis Reveal a Regulatory Module in Hepatic Lipid Metabolism Disorders in Mice With Subclinical Hypothyroidism. Exp. Ther. Med. 19, 897–906. doi: 10.3892/etm.2019.8281 32010250 PMC6966133

[B33] Zhou J. Dong X. Liu Y. Jia Y. Wang Y. Zhou J.. (2020). Gestational Hypothyroidism Elicits More Pronounced Lipid Dysregulation in Mice Than Pre-Pregnant Hypothyroidism. Endocr. J. 67, 593–605. doi: 10.1507/endocrj.EJ19-0455 32161203

[B34] Zhou L. Li X. Ahmed A. Wu D. Liu L. Qiu J.. (2014). Gut Microbe Analysis Between Hyperthyroid and Healthy Individuals. Curr. Microbiol. 69, 675–680. doi: 10.1007/s00284-014-0640-6 24969306

